# Applying mass spectrometry-based qualitative proteomics to human amygdaloid complex

**DOI:** 10.3389/fncel.2014.00080

**Published:** 2014-03-20

**Authors:** Joaquín Fernández-Irigoyen, María V. Zelaya, Enrique Santamaría

**Affiliations:** ^1^Clinical Neuroproteomics Group, Proteomics Unit, Navarrabiomed, Fundación Miguel ServetPamplona, Spain; ^2^Neurological Tissue Bank, Navarrabiomed, Fundación Miguel ServetPamplona, Spain

**Keywords:** brain, amygdala, proteomics, mass spectrometry, bioinformatics

## Abstract

The amygdaloid complex is a key brain structure involved in the expression of behaviors and emotions such as learning, fear, and anxiety. Brain diseases including depression, epilepsy, autism, schizophrenia, and Alzheimer's disease, have been associated with amygdala dysfunction. For several decades, neuroanatomical, neurophysiological, volumetric, and cognitive approaches have been the gold standard techniques employed to characterize the amygdala functionality. However, little attention has been focused specifically on the molecular composition of the human amygdala from the perspective of proteomics. We have performed a global proteome analysis employing protein and peptide fractionation methods followed by nano-liquid chromatography tandem mass spectrometry (nanoLC-MS/MS), detecting expression of at least 1820 protein species in human amygdala, corresponding to 1814 proteins which represent a nine-fold increase in proteome coverage with respect to previous proteomic profiling of the rat amygdala. Gene ontology analysis were used to determine biological process represented in human amygdala highlighting molecule transport, nucleotide binding, and oxidoreductase and GTPase activities. Bioinformatic analyses have revealed that nearly 4% of identified proteins have been previously associated to neurodegenerative syndromes, and 26% of amygdaloid proteins were also found to be present in cerebrospinal fluid (CSF). In particular, a subset of amygdaloid proteins was mainly involved in axon guidance, synaptic vesicle release, L1CAM interactome, and signaling pathways transduced by NGF and NCAM1. Taken together, our data contributes to the repertoire of the human brain proteome, serving as a reference library to provide basic information for understanding the neurobiology of the human amygdala.

## Introduction

The amygdaloid complex (also known as amygdala) is an almond-shaped brain structure located deep within the anterior portion of the temporal lobe (Schumann et al., 2011). Covering approximately 0.3% of the human brain volume (1–2 mm^3^ in mice), the amygdala is involved in processing emotions, olfactory memory, and social behaviors (Buchanan et al., 2003; Sah et al., 2003; Adolphs et al., 2005; Bickart et al., 2011; Fonslow et al., 2012). The amygdaloid complex consists of several anatomically and functionally distinct nuclei including the lateral amygdala, basal amygdala, and central nucleus. These are further divided into subdivisions covering at least 13 interconnected and intra-connected nuclei that can be distinguished both on cytoarchitectonic and connectional areas, having afferent and efferent connections with cortical and subcortical regions (Pitkanen et al., 1997, 2003; Sah et al., 2003). The basolateral structures consist of a majority of glutamatergic projection neurons and a minority of GABAergic interneurons, while medial nuclei are striatum-like, with a majority of medium-sized spiny GABAergic neurons (Ehrlich et al., 2009).

Amygdala dysfunction has been implicated in the symptomatology of pathological conditions including depression, epilepsy, Alzheimer's disease, autism, and schizophrenia (Boccardi et al., 2002; Aroniadou-Anderjaska et al., 2008; Bellani et al., 2011; Schumann et al., 2011). In view of these data, an in depth biochemical characterization of the normal amygdala is mandatory as a first step for understanding the role of the amygdala in neuropathology. Therefore, there is an increasing interest in the identification of molecular mediators orchestrating the amygdaloid complex functionality. Transcriptional profiling studies have been conducted to examine the genomic fingerprint of the amygdala subnuclei (Dent et al., 2001; Zirlinger et al., 2001; Becker et al., 2008). Moreover, previous studies have partially characterized the lipidome and metabolome present in the amygdala using mass spectrometry techniques (Cheng et al., 2011; Gonzalez et al., 2011). However, proteomics is expected to provide a more extensive description of the molecular substrates involved in normal amygdala function. Specifically, MS-based qualitative proteomics has been employed to profile the murine amygdala proteome and phosphoproteome (Katagiri et al., 2010; Fonslow et al., 2012). On the other hand, quantitative proteomics based on a combination of 2-DE or stable isotopic tags with MS has been used to describe protein and peptide profiles from amygdala in several murine models (Berezniuk et al., 2010; Wardman et al., 2010; Whittle et al., 2011; Hong et al., 2013) and also to characterize the amygdala sensitization against different chemical insults such as ethanol (Bell et al., 2006), metamphetamine (Iwazaki et al., 2008), nicotine (Hwang and Li, 2006), morphine (Lin et al., 2011), and opiate (Zill et al., 2011). However, despite these efforts to identify and catalog part of the altered proteins present in the amygdaloid region of these genetically-modified and chemical murine models, only a very limited number of proteins have been mapped in human amygdala (Kekesi et al., 2012).

To further advance a comprehensive understanding of amygdala biology, we used protein and peptide fractionation strategies coupled to nanoLC-MS/MS to perform a proteomic characterization of the human amygdaloid complex in depth, and present the first large-scale characterization of this brain substructure. We report the identification of 1820 protein species in the amygdala derived from three healthy subjects. We provide a brief overview of molecular functions and subcellular localizations of identified proteins based on Gene Ontology analysis. Extensive database analysis revealed that 4% of the identified proteins were previously associated with neurodegenerative diseases and more than 60% of the identified proteins have not been previously reported in proteome descriptions of human limbic system structures. This high-confidence collection of proteins present in human amygdala provides fundamental information for the ultimate characterization of amygdala function in human brain.

## Materials and methods

### Sample collection

According to the Spanish Law 14/2007 of Biomedical Research, inform written consent form of the Neurological Tissue Bank of Navarra Health Service was obtained for research purposes from relatives of the 3 European patients included in this study. All subjects were male and ages ranged from 41 to 61 years. All assessments, *post-mortem* evaluations, and procedures were previously approved by the Clinical Ethics Committee of Navarra Health Service. According to standard practices in place at the neurological tissue banks, the left cerebral hemisphere was progressively frozen, sliced into 1–1.5 cm-thick coronal sections and stored at −80°C until used (*post-mortem*-interval: 4–8 h). Therefore, the amygdala assessed in this study was the left one. The diagnosis was carried out on the right cerebral hemisphere. Following fixation in 10% formaldehyde for approximately 3 weeks, the brains was sectioned according to the recommendation guide proposed by BrainNet Europe (Bell et al., 2008). After a macroscopic study, immunohistochemistry analysis was performed in different brain regions using specific antibodies against Tau protein, β amyloid, TDP-43, PrP, α-synuclein, ubiquitin, and α−β crystalline. These brains did not show significant pathology and were considered to be healthy.

### Sample preparation for proteome analysis

Basolateral amygdala specimens were obtained from frozen brain sections using sterile biopsy punches (size: 3–4 mm) and homogenized in lysis buffer containing 7 M urea, 2 M thiourea, 4% (v/v) CHAPS, 50 mM DTT. The homogenates were spinned down at 100.000 × g for 1 h at 15°C. Protein concentration was measured in the supernatants with the Bradford assay kit (Bio-Rad). Prior to proteomic analysis, the amygdala samples were pooled (~200 μg of protein from each specimen).

### Peptide fractionation by HPLC

Protein material (~350 μg of protein) was precipitated using methanol/chloroform extraction. Pellet was dissolved in 6 M urea, 100 mM Tris, pH 7.8. Reduction was performed by addition of DTT to a final concentration of 10 mM and incubation at 25°C for 1 h. Subsequent alkylation by 30 mM iodoacetamide was performed for 1 h in the dark. An additional reduction step was performed by 30 mM DTT, allowing the reaction to stand at 25°C for 1h. Proteins were digested for 4 h with Lys-C (Promega) at 37°C (enzyme:protein, 1:140 w/w). The mixture were then diluted to 0.6 M urea using MilliQ-water, and after trypsin addition (Promega) (enzyme:protein, 1:50 w/w), the sample was incubated at 37°C for 18 h. Digestion was quenched by acidification with acetic acid. The digestion mixture was dried in a SpeedVac, reconstituted with 40 ul of 5 mM ammonium bicarbonate (ABC) pH 9.8, and injected to an Ettan LC system with a high pH stable X-Terra RP18 column (C18; 2.1 mm × 150 mm; 3.5 μm) (Waters) at a flow rate of 40 μl/min. Peptides were eluted with a mobile phase B of 5–65% linear gradient over 35 min (A, 5 mM ABC in water at pH 9.8; B, 5 mM ABC in acetonitrile at pH 9.8). Twenty fractions were collected (Supplementary Figure 1), evaporated under vacuum and reconstituted into 15 μl of 2% acetonitrile, 0.1% formic acid, 98% MilliQ-H_2_0 prior to mass spectrometric analysis.

### Protein fractionation by isoelectric focusing (IEF)

Approximately 150 μg of pooled sample was precipitated with methanol/chloroform. The pellet was re-suspended in 300 μl of IEF rehydration buffer (7 M urea, 2 M thiourea, 2% CHAPS, 50 mM DTT, 0.5% Bio-Lyte 3/10 ampholyte) and loaded on 17 cm pH 3–10 nonlinear immobilized pH gradient strip in a focusing tray. IEF was performed on a Bio-Rad PROTEAN IEF system (Bio-Rad). Conditions for performing IEF were as follows: after rehydration of the IPG strip for 12 h at 50 V and 20°C, the run was started at 250 V for 15 min followed by rapid voltage ramping to 10,000 V without exceeding 50 A/strip. The IEF run was finished when the voltage reached 60,000 V. The voltage was held at 500 V until the run was stopped. The IEF run was performed at 20°C. The entire IPG strip was divided into 25 equal parts (~0.7 cm each) for in-gel reduction, alkylation, and digestion. In-gel tryptic digestion was performed with 20 ng/μl trypsin in 50 mM ABC for 18 h at 37°C. The resulting tryptic peptides were extracted with 5% formic acid, 50% acetonitrile, and 5% formic acid, 85% acetonitrile. Peptide mixtures were completely dried in a SpeedVac and resuspended in 20 μl 98% MilliQ-H_2_0 2% acetonitrile 0.1% formic acid.

### Mass spectrometry analysis

For each fraction, a total volume of 5 μl of tryptic peptides was injected with a flow rate of 300 nL/min in a nanoLC Ultra1D plus (Eksigent). The column and the autosampler were maintained at a temperature of 40°C and 4°C respectively. A trap column Acclaim PepMap100 (100 μm × 2 cm; C18, 5 μm, 100 Å) and an analytical column Acclaim PepMap RSLC (75 μm × 15 cm, C18, 2 μm, 100 Å) (Dionex) were used following the next gradient: 0–1 min (2% Buffer B), 1–110 min (2–30% Buffer B), 110–120 min (30–40% Buffer B), 120–125 min (40–90% Buffer B), 125–130 min (90% Buffer B), 130–132 min (90–2% Buffer B) and 132–150 min (2% Buffer B) [Buffer B (100% acetonitrile, 0.1% formic acid), Buffer A (0.1% formic acid)]. MS analysis was performed on a Q-TRAP 5500 system (ABSciex) with a NanoSpray® III ion source (ABSciex). The mass spectrometer was operated in positive ion mode at unit resolution. Each fraction was analyzed twice in technical replicates (90 LC-MS/MS runs). For MS analysis, Enhanced MS (EMS) and Enhanced Resolution (ER) scans were acquired at a scan rate of 10,000 Da/s and 250 Da/s respectively. The ER scan was used to confirm the charge state and confirm the mass assignment. For MS/MS analysis, survey scans were acquired from m/z 400 to 1000 with up to 6 precursors selected for MS/MS (Enhanced Product Ion scan) from m/z 230 to 1000 using dynamic exclusion, and the rolling collision energy was used to promote fragmentation. MS/MS data acquisition was performed using Analyst 1.5.2 (AB Sciex) and submitted to Protein Pilot™ Software (v.4.0.8085-ABSciex) using Paragon™ Algorithm (v.4.0.0.0) (Shilov et al., 2007) for database search restricted to *Homo Sapiens* (Database: uniprot_sprot_20100622; Unused Protein score greater than 1.3, corresponding to a 95% confidence). False discovery rate (FDR) was performed using a nonlinear fitting method (Tang et al., 2008) and displayed results were those reporting a protein level-FDR lower than 1%. Only proteins identified with ≥2 peptides were considered for further analysis.

### Data handling and bioinformatic analysis

The proteins identified in this study were classified by DAVID (Database for Annotation, Visualization, and Integrated Discovery) Bioinformatics Resources (v6.7) (Huang Da et al., 2009), where proteins are assigned in gene ontology (GO) terms, which rely on a controlled vocabulary for describing a protein in terms of its molecular function, biological process, or subcellular localization (Ashburner et al., 2000). For functional annotation clustering, we set the following parameters: “Biological process,” high stringency and EASE *p* < 0.01; “molecular Function,” high stringency and EASE *p* < 0.05. The hydrophobicity score of plasma membrane proteins (GRAVY Index) was calculated using the ProtParam tool at Expasy Server (http://web.expasy.org/protparam/). Reactome Database (http://www.reactome.org) (Haw et al., 2011) was used to perform overrepresentation analysis of human amygdala proteins across specific reactions, in order to recognize those biological pathways likely to be active in human amygdala. The significance of the association between the protein list and a certain pathway was expressed by a *p*-value, expressing the probability (hypergeometric test) that the association between the amygdala proteins and a specific pathway is explained by chance alone (only *p* < 0.01 were considered significant).

## Results

### Identification of human amygdaloid proteins by protein and peptide separation strategies coupled to mass spectrometry

**Figure 1:**
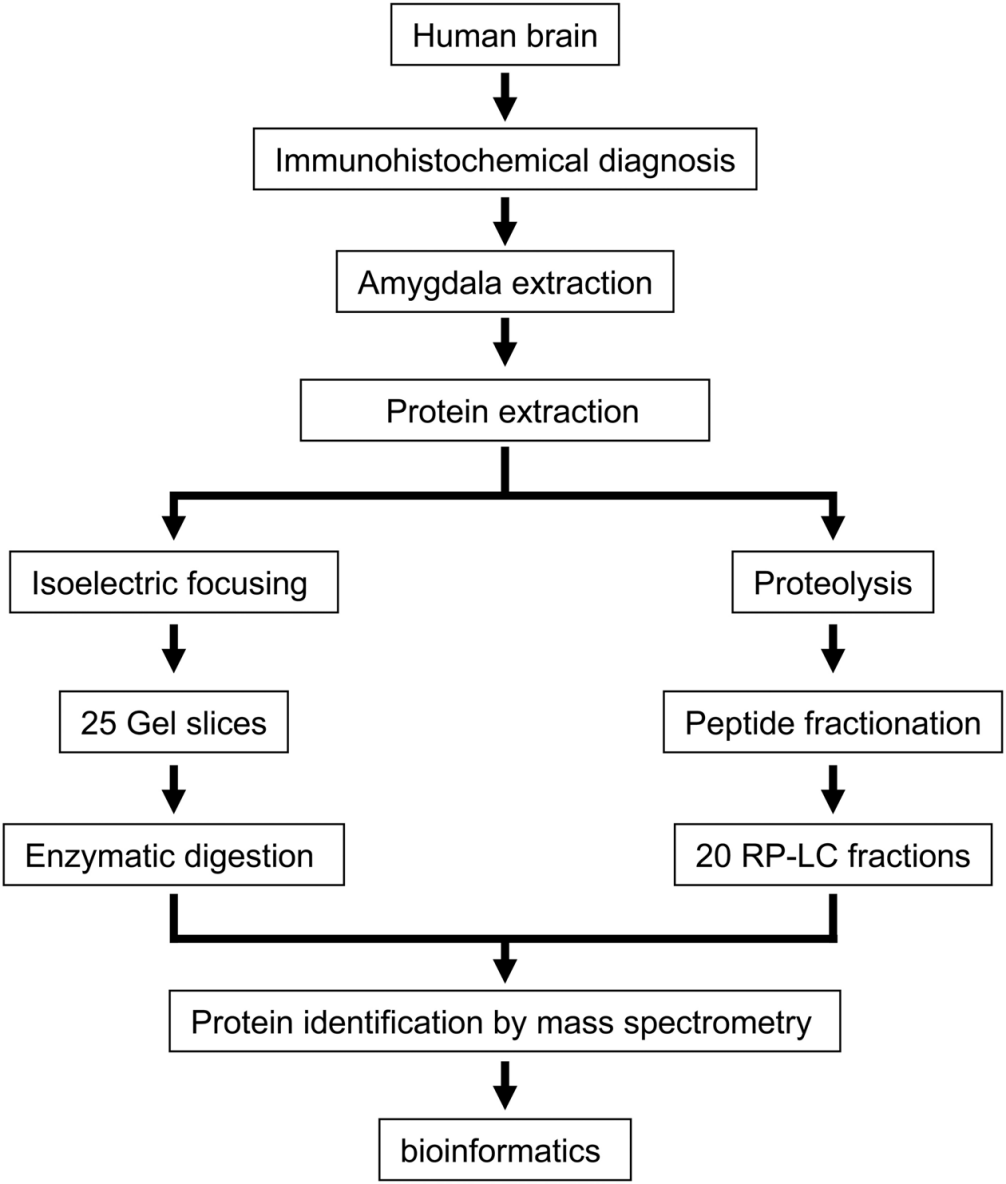
**An overview of the workflow used for identification of the amygdaloid proteome**.

In the present study, we have used autopsy specimens of the left basolateral amygdala from three healthy human brains with the final goal to obtain a profound insight into the protein content and protein function of the amygdaloid complex. To reduce protein complexity, we used an integrated experimental workflow combining IEF and chromatographic-based methods coupled to mass spectrometry (Figure 1). First, proteins were separated by IEF and the gel was sliced in 25 portions followed by in-gel trypsin digestion. The second approach involved in- solution digestion followed by off-line RP-LC at basic pH to separate the peptide mixture in 20 fractions. Replicate mass spectrometry measurements were performed in all peptide fractions. Combining both approaches, we have generated an amygdala proteome map of 1820 protein species with a median sequence coverage of 23% (95% confidence), identified with a FDR lower than 1%. Complete lists of identifications and their corresponding scores are presented in Supplementary Tables 1 and 2 in http://figshare.com/account/my_data

**Figure 2:**
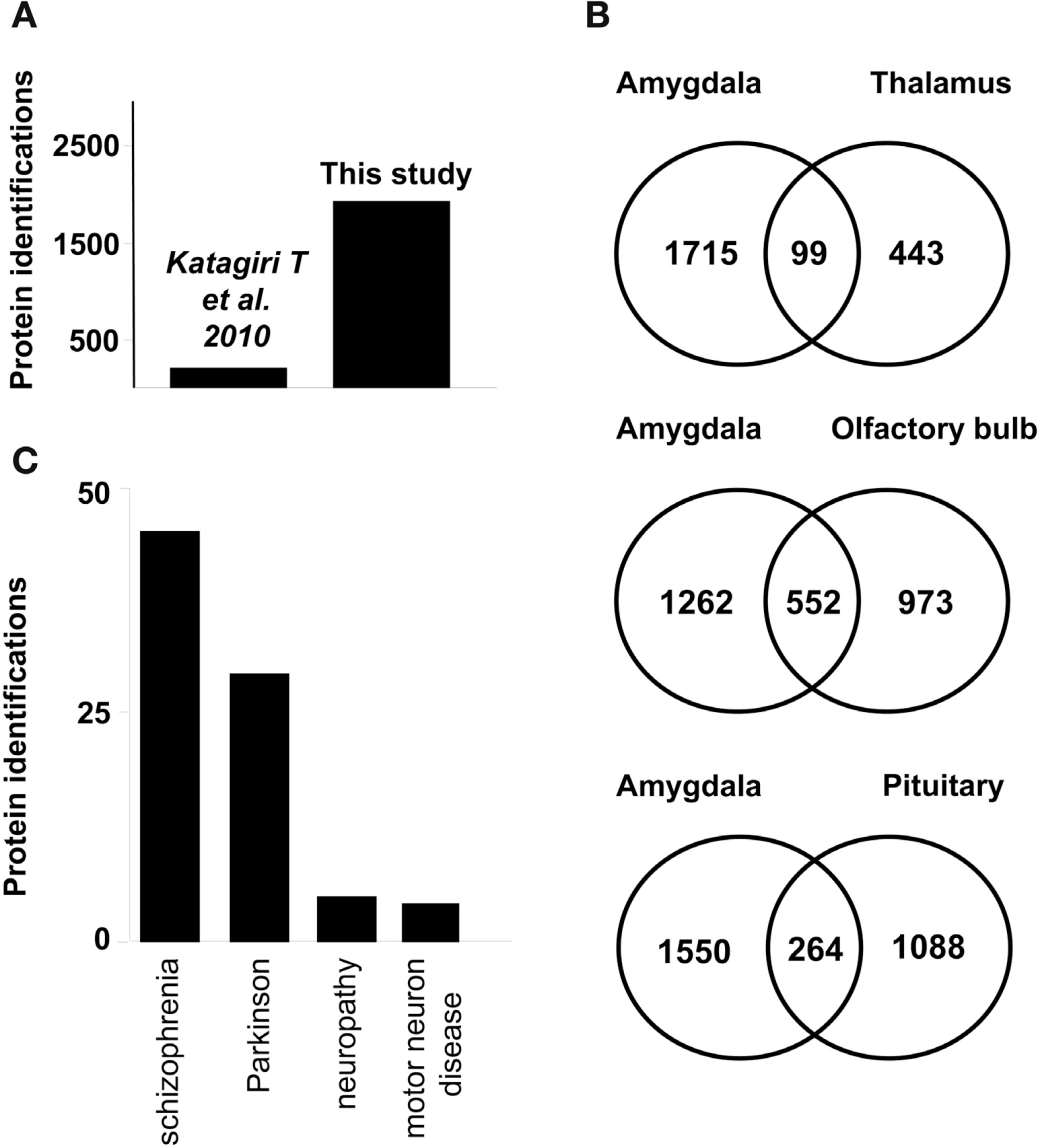
**(A)** Graph showing the increase in identifications from a previous study achieved in rat amygdala **(B)** Venn diagrams of proteins found in human limbic system proteome datasets. Numbers represent the number of shared proteins in the respective overlapping areas. Human proteins reported in thalamus (Martins-de-Souza et al., 2009) (upper) Human proteins reported in OB (Fernandez-Irigoyen et al., 2012) (middle) Human proteins reported in pituitary tissue (Krishnamurthy et al., 2011) (lower). **(C)** Graph showing the number of proteins associated with neurodegenerative syndromes according to Genetic Association Database.

Protein identification data from the current study were compared with previously published dataset of murine amygdala proteome. As shown in Figure 2A, our dataset represents a nine-fold increase in proteome coverage with respect to previous amygdala proteome description from rat model (Katagiri et al., 2010). On the other hand, our dataset was compared with previous proteomic descriptions derived from large-scale proteomic studies of limbic system structures such as human thalamus (Martins-de-Souza et al., 2009), human olfactory bulb (Fernandez-Irigoyen et al., 2012), and human pituitary gland (Krishnamurthy et al., 2011), showing a 18, 36, and 19,5% overlapping respectively (Figure 2B). According to Genetic Association Database (Becker et al., 2004), 65 amygdaloid proteins identified in this study have been previously linked to neurodegenerative syndromes such as schizophrenia, and Parkinson's diseases (Figure 2C and Supplementary Table 3).

### Human amygdaloid proteome characterization functional metrics

**Figure 3:**
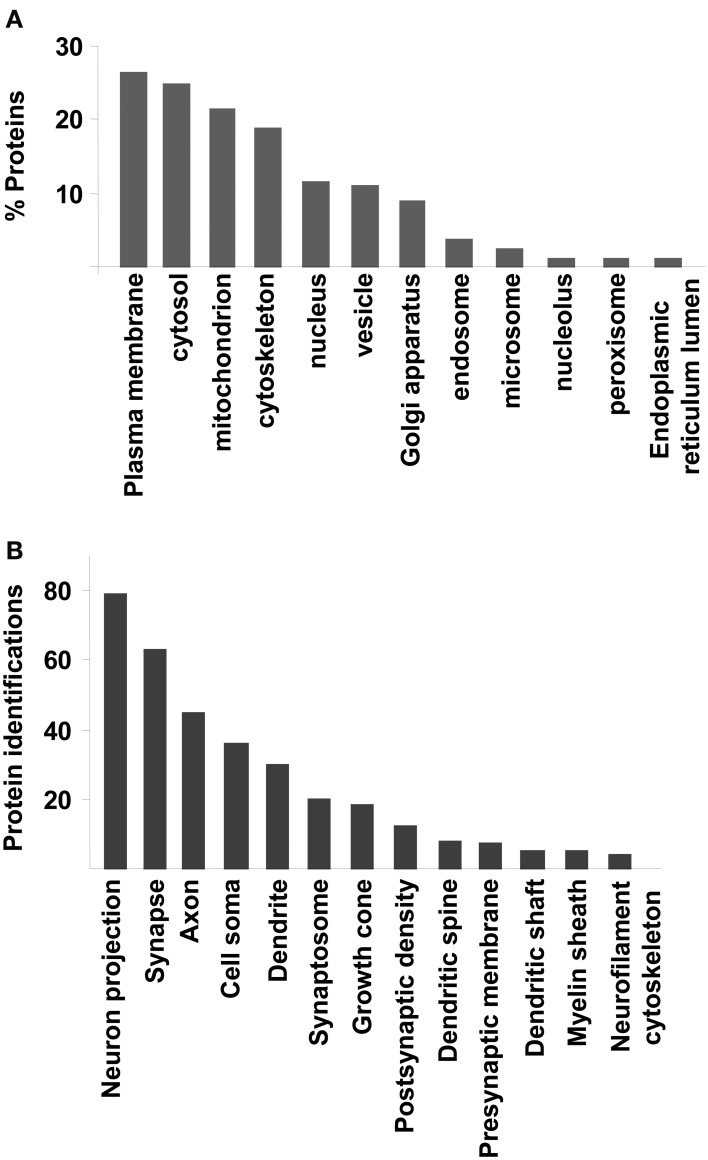
**Cellular Component Ontology. (A)** classification of amygdaloid proteome based on cellular localization. **(B)** Neuron-specific localization detected by DAVID software.

To extract biological knowledge, the amygdaloid proteome dataset was functionally categorized based on Gene Ontology (GO) annotation code using DAVID software (Huang Da et al., 2009). From our dataset, 1312 identifiers were considered for further analysis. 1201 (91,5%), 1159 (88,3%), and 1229 (93,6%) proteins were linked to at least one annotation term within the GO cellular component, biological process, and molecular function categories respectively. As shown in Figure 3, 26% was accounted for plasma membrane proteins. Another significant proportion of the identified proteins consisted of cytosolic (24%), mitochondrial (21%), cytoskeletal (18%), nuclear (11%), and vesicle proteins (11%) (Figure 3A). GO hierarchy also showed that 9% of amygdaloid proteins was associated to Golgi apparatus. The remaining assignments included proteins present in endosomes (3,5%), microsomes (2%), and nucleolus (1%) between others (Figure 3A and Supplementary Table 4). The Grand Average of Hydropathicity Index (GRAVY index) was calculated for the 316 proteins annotated as plasma membrane proteins where 30 proteins (10%) had positive values (hydrophobic proteins) (Supplementary Table 4). A neuron-specific cell component analysis was also performed, detecting proteins associated to neuron projection (6,5%), synapse (5%), axon (4%), cell soma (3%), dendrite (2,5%), and synaptosome (2%) between others (Figure 3B and Supplementary Table 4).

**Figure 4:**
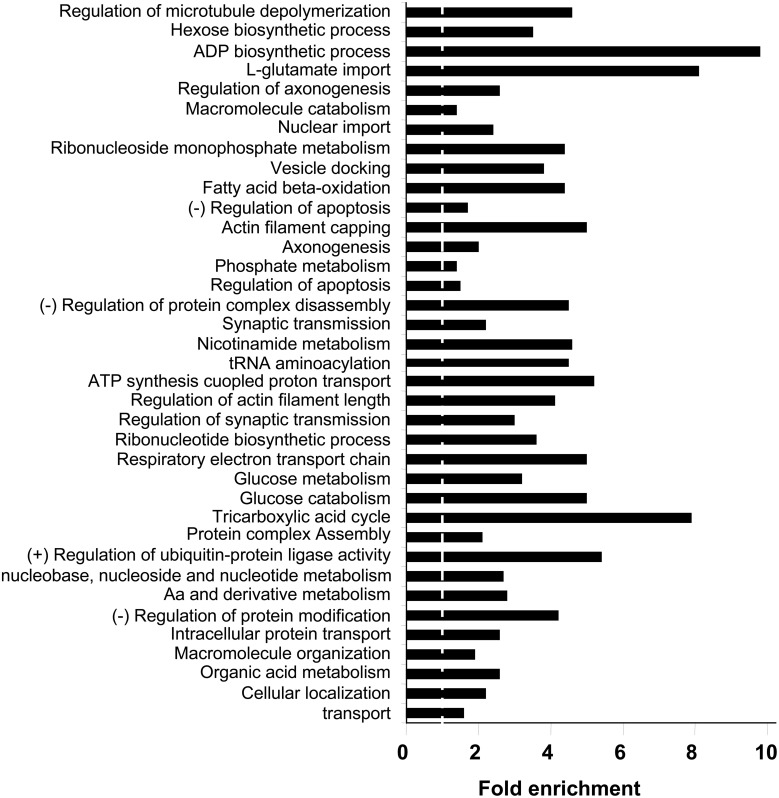
**Biological Process Ontology for the amygdala proteomic expression profile**. Representative enriched GO biological process terms from 37 significantly annotation clusters are shown (EASE *p* < 0.01). Fold enrichment refers to the number of relevant amygdaloid protein species represented in each category relative to random expression of all genes in the human genome. A complete characterization of each cluster is shown in Supplementary Table 5.

With respect to the biological process and molecular function categories, an enrichment analysis has been performed against *Homo Sapiens* background using functional annotation clustering provided in DAVID 6.7 software. Respects to biological process ontology, 37 clusters are significantly enriched in human amygdala respect to human genome (Supplementary Table 5). Some of the most significantly enriched biological processes included transport (*P*-value: 5,1E-24), cellular localization (*P*-value: 4,8E-22), organic acid metabolism (*P*-value: 1,2E-22), cellular aminoacids and derivative metabolism (*P*-value: 1,2E-17), and nucleobase, nucleoside, and nucleotide metabolism (*P*-value: 8,2E-14). Representative biological process categories from each cluster are shown in Figure 4. A complementary analysis of biological processes was performed with a search of KEGG pathways (Kanehisa and Goto, 2000) that are over-represented in human amygdala. In this case, 621 out of 1312 identifiers (47%) were mapped to KEGG pathways. The 24 pathways represented with a high statistical significance (fold enrichment >1.5; EASE *p* < 0.01) are shown in Supplementary Table 6. Three of these pathways are clearly related to neurological disorders such as Parkinson's, Alzheimer's, and huntington's diseases (see amygdaloid proteins mapped in these KEGG pathways in Supplementary Figures 2–4). Among the KEGG pathways, oxidative phosphorylation, proteasome, and aminoacyl-tRNA biosynthesis, are of particular interest because they confirm the significant enrichment of pathways involved in mitochondrial energy generation as well as in protein synthesis and degradation. Strong representation of carbohydrate, amino acid, and lipid metabolism, together with the regulation of neurological system process such as lon-term potentiation and endocytosis, parallels with previous observations that the biological process GO Terms “glucose metabolism/catabolism,” Cellular aminoacid metabolism,” “Fatty acid beta oxidation,” “regulation of synaptic transmission,” and “vesicle docking” are highly enriched in human amygdala.

**Figure 5:**
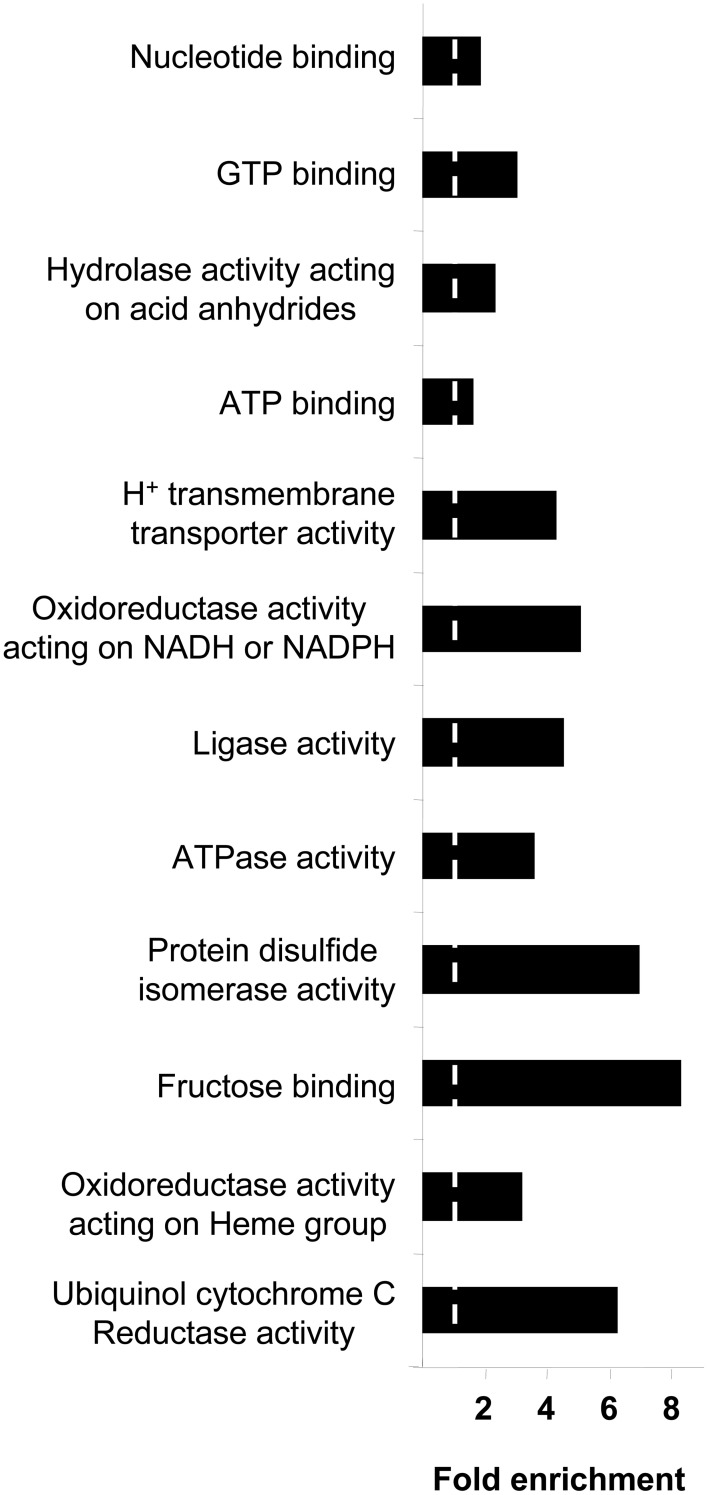
**Molecular Function Ontology for the amygdala proteomic expression profile**. Representative enriched GO molecular function terms from 12 significantly annotation clusters are shown (EASE *p* < 0.05). Fold enrichment refers to the number of relevant amygdaloid protein species represented in each category relative to random expression of all genes in the human genome. A complete characterization of each cluster is shown in Supplementary Table 7.

Respects to molecular function ontology, 12 clusters are significantly enriched in our human amygdala dataset (Supplementary Table 7). GTP binding (*P*-value: 9,0E-21), nucleotide binding (*P*-value: 2,4E-32), hydrolase activity (*P*-value: 7,9E-21), and ATP binding (*P*-value: 7,9E-11) were the most significantly enriched molecular functions in our dataset. Representative molecular function categories from each cluster are represented in Figure 5. Subsequent analyses were performed to analyse the amygdaloid protein dataset distribution across specific biological reactions using curated Reactome Database. 688 out of 1814 amygdaloid proteins were mapped to 464 biological reactions (Supplementary Table 8) being proteasome mediated degradation, gene expression, metabolism, and homeostasis the general over-represented processes. Interestingly, as shown in Table 1, some statistically over-represented processes were directly relevant to neurotransmitter release, electrical machinery, and synaptic plasticity.

**Table 1:** **Over-representation of human amygdaloid proteins in specific-neuronal processes by Reactome pathway analysis**.

**Name of the event**	**Amygdaloid proteins in each event**	**Total number of proteins in each event**	***p*-value**
Axon guidance	67	277	1.0e–12
L1CAM interactions	37	107	2.2e–12
Transmission across chemical synapses	45	191	1.6e–08
GABA synthesis, release, reuptake, and degradation	12	19	2.0e–08
Release of GABA at the synapse	9	13	4.2e–07
GABA loaded synaptic vesicle docking and priming	9	13	4.2e–07
Membrane trafficking	41	192	1.2e–06
Serotonin neurotransmitter release cycle	8	12	3.0e–06
Dopamine neurotransmitter release cycle	8	12	3.0e–06
Dopamine synaptic vesicle docking and priming	8	12	3.0e–06
Release of docked dopamine loaded synaptic vesicle	8	12	3.0e–06
Release of docked serotonin loaded synaptic vesicle	8	12	3.0e–06
Serotonin loaded synaptic vesicle docking and priming	8	12	3.0e–06
Trafficking of AMPA receptors	12	30	1.2e–05
Glutamate binding, activation of AMPA receptors and synaptic plasticity	12	30	1.2e–05
Assembly in clathrin-coated vesicles (CCVs)	9	18	1.8e–05
Opioid Signaling	21	80	2.0e–05
Formation of clathrin coated vesicle	7	11	2.0e–05
Loading of GABA into clathrin sculpted GABA transport vesicle lumen	6	8	2.1e–05
Trafficking of GluR2-containing AMPA receptors	8	16	5.4e–05
Retrograde neurotrophin signaling	7	13	8.8e–05
Neuronal system	48	292	2.3e–04
Sema3A PAK dependent axon repulsion	7	15	2.8e–04
Trafficking of GluR2-containing AMPA receptors to extrasynaptic sites	7	15	2.8e–04
Norepinephrine neurotransmitter release cycle	6	12	4.9e–04
Endocytosis of Ca impermeable AMPA receptors	6	12	4.9e–04
Axonal growth inhibition (RHOA activation)	5	9	8.2e–04
Glutamate synaptic vesicle docking and priming	5	9	8.2e–04
release of L-glutamate at the synapse	5	9	8.2e–04
Acetylcholine synaptic vesicle docking and priming	5	9	8.2e–04
Release of acetylcholine at the synapse	5	9	8.2e–04
Release of noradrenaline at the synapse	5	9	8.2e–04
Noradrenalin synaptic vesicle docking and priming	5	9	8.2e–04
Neurofascin binds contactin-1:CASPR complex	3	3	9.5e–04
AGRN binds NCAM1, PTPRS	3	3	9.5e–04
Axonal transport of NGF:Trk complexes	5	10	1.5e–03
p75NTR regulates axonogenesis	5	10	1.5e–03
Regulation of Insulin Secretion by acetylcholine	5	10	1.5e–03
Glutamate neurotransmitter release cycle	6	15	2.1e–03
NCAM signaling for neurite out-growth	15	68	2.1e–03
Acetylcholine neurotransmitter release cycle	5	11	2.6e–03
NCAM1:pFAK:Grb2:Sos-mediated nucleotide exchange of Ras	6	16	3.0e–03
NGF signaling via TRKA from the plasma membrane	32	201	4.1e–03
Signaling by NGF	42	283	4.2e–03
Unblocking of NMDA receptor	6	17	4.3e–03
Unblocking of NMDA receptor, glutamate binding, and activation	6	17	4.3e–03
DARPP-32 events	7	24	6.8e–03
Sema4D induced cell migration and growth-cone collapse	7	24	6.8e–03
Neurotransmitter receptor binding. Transmission in the post-synaptic cell	23	137	7.4e–03
Activation of NMDA receptor upon glutamate binding and post-synaptic events	9	37	8.3e–03
glutamate uptake by astrocytes	2	2	9.7e–03
Interaction of NCAM1 with Neurocan	2	2	9.7e–03
Interaction of NCAM1 with major prion protein (PrP)	2	2	9.7e–03
Interaction of NCAM1 with agrin	2	2	9.7e–03
Enzymatic degradation of dopamine by COMT	2	2	9.7e–03
Enzymatic degradation of dopamine by monoamine oxidase	2	2	9.7e–03
Metabolism of serotonin	2	2	9.7e–03
Serotonin clearance from the synaptic cleft	2	2	9.7e–03
Degradation of GABA	2	2	9.7e–03

*P-value indicates the probability that the association between amygdaloid proteins and the molecular event is explained by chance (only p < 0.01 were considered). See Supplementary Table 8 to show the specific proteins in each event*.

### Localization of human amygdaloid proteins in CSF

**Figure 6:**
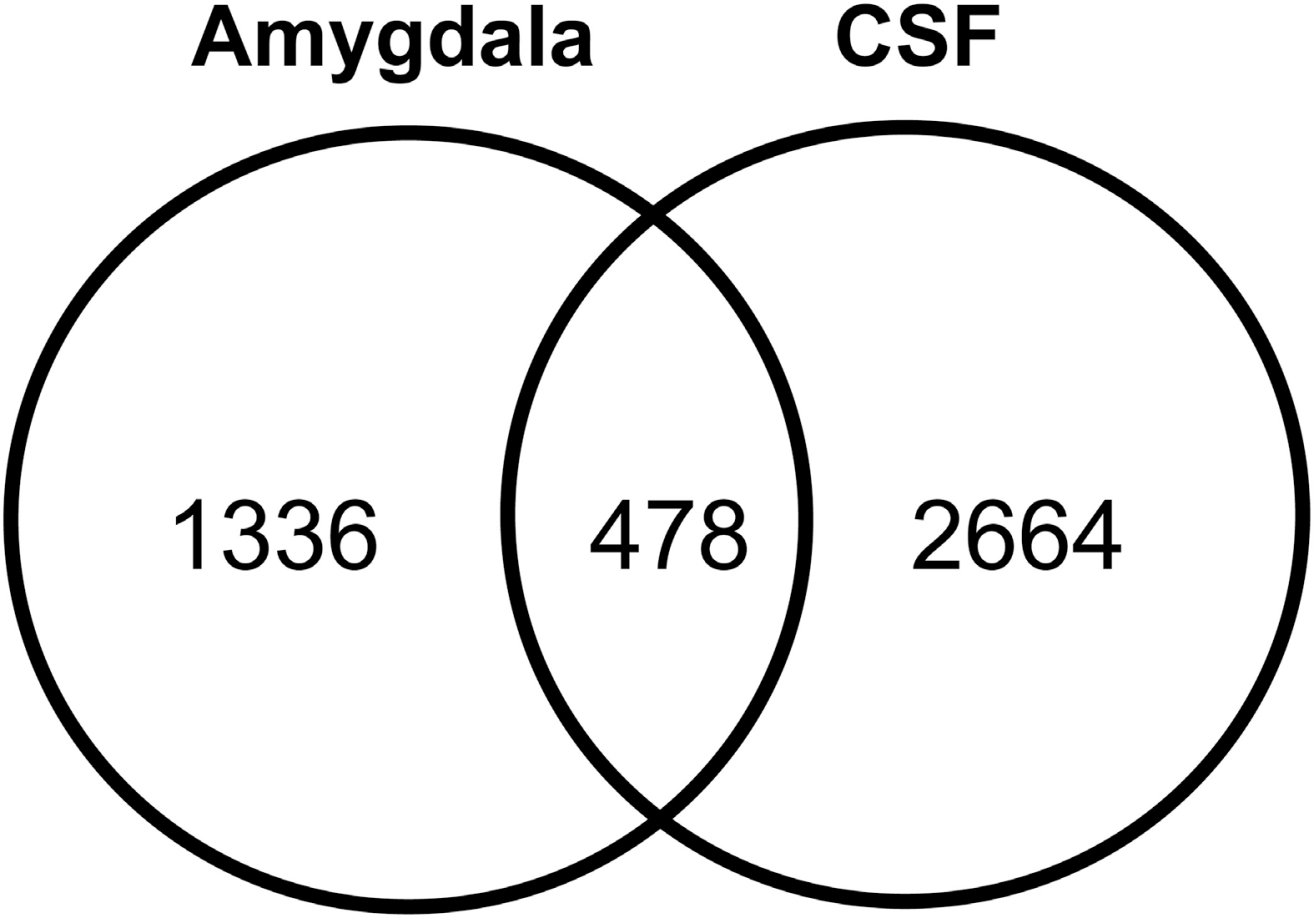
**Overlap of amygdaloid complex proteome characterized in this study with CSF proteome datasets (Pan et al., 2007b; Schutzer et al., 2010)**.

The human amygdaloid proteome dataset was also compared with previously published lists of human CSF proteome descriptions (Pan et al., 2007b; Schutzer et al., 2010). Four hundred and seventy eight proteins (26%) were found to exist in both the amygdaloid and CSF proteomes (Figure 6). A subset of these proteins is known to be involved in a plethora of central nervous system functions and some of them are implicated in the development of a few neurological diseases such as amyotrophic lateral sclerosis, Parkinson's and Alzheimer's diseases (Supplementary Table 9).

## Discussion

Amygdala dysfunction has been implicated in pathological conditions including depression, schizophrenia, and Alzheimer's disease between others (Boccardi et al., 2002; Aroniadou-Anderjaska et al., 2008; Bellani et al., 2011; Schumann et al., 2011). Considering that the identification of standard amygdala proteome involved in normal physiology will contribute to the delineation of neurodegenerative diseases mechanisms, there is an urgent need for identifying and categorizing all proteins responsible for maintaining normal amygdala function. The objective of this study was to gain new insight into the whole protein composition of the human amygdaloid complex. Since it is well described that usage of different fractionation techniques enhances the dynamic range of the experimental setup and increases proteome coverage (Qian et al., 2006), we have performed an in-depth analysis of the protein content of healthy human basolateral amygdala using complementary protein and peptide fractionations methods coupled to tandem mass spectrometry, identifying 1814 unique proteins from which 4% are associated to neurodegenerative syndromes. To our knowledge, this is the first large-scale qualitative proteomic analysis for human amygdaloid complex up to now. Interestingly, this dataset increases significantly the catalog of proteins identified in human limbic system so far, revealing more than 60% of the identified proteins not previously described before in thalamus, olfactory bulb, or pituitary gland (Martins-de-Souza et al., 2009; Krishnamurthy et al., 2011; Fernandez-Irigoyen et al., 2012). Similar experimental workflows have been successfully applied in proteome characterization of different human brain subproteomes such as cortex and olfactory bulb (Frohlich et al., 2006; Fernandez-Irigoyen et al., 2012). In our case, we have used three independent autopsy specimens from the left amygdala with a *post-mortem* interval (PMI) between 4 and 8 h, where proteins are reasonably stable (Hynd et al., 2003; Crecelius et al., 2008; Ferrer et al., 2008).

Encompassing the 90 LC-MS/MS runs performed in both separative approaches, a nonredundant set of 1814 unique proteins were identified with median sequence coverage of 23%. Although our study has uncovered many intricacies in protein expression in amygdaloid complex, there are potential limitations of our study that warrant discussion. We have used a Q-TRAP instrument for mass spectrometry analysis (Hopfgartner et al., 2004), identifying highly abundant proteins and housekeeping enzymes present in human amygdala. Future studies employing high-resolution instruments (Mann and Kelleher, 2008; Andrews et al., 2011) will increase the quality of amygdala proteome data in terms of high resolving power, mass accuracy, and high sequencing speed, generating novel proteomic data with high impact from a functional point of view. Although all individuals were males of similar age and ethnicity, our data do not capture population or sex diversity. Taking into account that human brain transcriptome is highly dynamic during neurodevelopment (Kang et al., 2011) additional proteomic studies employing post-mortem brains of clinically unremarkable donors representing males and females with different ages and ethnic backgrounds are necessary to estimate the consistency of the proteomic profile obtained in this study.

In view of the importance of the dynamics and constant remodeling of the microtubule cytoskeleton during axonal growth cone (Estrada-Bernal et al., 2012), we expected and found a high proportion (18%) of proteins involved in the regulation of microtubule cytoskeleton where cytoplasmic dynein 1 heavy chain, spectrin beta-nonerythrocytic 1, isoform 2 of spectrin alpha chain, and isoform 7 of plectin were identified with more than 100 peptide species. In agreement with intracellular distribution of human proteins in several brain structures (Mueller et al., 2006; Fernandez-Irigoyen et al., 2012), a high proportion of proteins identified in amygdala correspond to plasma membrane proteins with a clear enrichment of cytosolic, nuclear, mitochondrial proteins and proteins associated to cytoskeleton. Similar subcellular distribution has been also described in human skeletal muscle proteome (Yi et al., 2008) in contrast with the distribution observed in proteomic studies derived from metabolic tissues such as heart and the liver where most of the assignments are directly related to organelles (Ruse et al., 2004; Chen et al., 2007; Bousette et al., 2009).

One of the goals of the present study was to generate extensive and robust data on the functional groups of proteins present in human amygdala. For this purpose, we have used a functional annotation clustering tool available in DAVID software. This type of grouping of functional annotation is able to give a more insightful view of the relationships between annotation categories and terms compared with the traditional linear list of enriched terms (Huang Da et al., 2009). However, a significant number of identified amygdaloid proteins have unknown functions. This is consistent with the data obtained in human cortex and thalamus where also more than 20% of the proteins identified by shotgun methods could not be assigned to biological processes (Pan et al., 2007a; Martins-de-Souza et al., 2008, 2009). In agreement with different brain proteomic studies (Pan et al., 2007a; Fernandez-Irigoyen et al., 2012), a high proportion of identified proteins presents catalytic and binding activities, suggesting that both functional protein groups may correspond to highly abundant proteins and tend to represent the majority of proteins identified in unfractionated neural tissue. In accordance with other brain proteomic studies (Mueller et al., 2006; Fernandez-Irigoyen et al., 2012), a significant enrichment of proteins with GTP binding activity was detected in human amygdala. In particular, members of the Rho GTPase inhibitors (GTPase activating proteins), activators (guanine nucleotide exchange factors), and Rab GTPase activating proteins were identified. These families of proteins are involved in neurite outgrowth, dendritic spine formation, axonogenesis, and synaptic vesicle exocytosis (Govek et al., 2005; Ng and Tang, 2008; Hall and Lalli, 2010; Tolias et al., 2011). Twelve RNA binding proteins correspond to heterogeneous nuclear ribonucleoproteins (HnRNPs) were also identified in this study. This family of proteins is involved in RNA trafficking, binding to A2RE-containing neuronal RNAs that move along dendrites in response to synaptic activities related to memory formation (Smith, 2004) and regulates the expression of specific proteins such as myelin basic protein, calcium-calmodulin-dependent protein kinase II alpha subunit or neurogranin (Smith, 2004; Kosturko et al., 2006). Using manually curated Reactome Database (Haw et al., 2011), we have obtained a deeper functional analysis of the amygdala dataset, detecting a plethora of neuronal molecular events enriched in human amygdala. Nearly forty interactors of neural cell adhesion molecules (L1CAM and NCAM1) were identified in our dataset. Although both molecules have long been involved in synaptic transmission and plasticity via intracellular signaling (Dityatev et al., 2008), recent studies demonstrate that they act as coreceptors of integrins and growth factors for repellent axon guidance molecules (Schmid and Maness, 2008). On the other hand, Reactome pathway analysis also pointed out the presence of 42 amygdaloid protein intermediates of signaling pathway transduced by nerve growth factor (NGF), a pivotal molecule that regulates neuronal survival, axonal growth, and synaptic plasticity (Lykissas et al., 2007).

By examining proteins previously identified in human CSF (Pan et al., 2007b; Schutzer et al., 2010), 478 amygdaloid proteins were also detected in this fluid, accounting for 26% of 1814 detected proteins. Further analysis have revealed that a subset of these proteins are known to be specifically involved in Parkinson's and Alzheimer's diseases. From the point of view for biomarker discovery, it is crucial to identify the presence of brain tissue analytes in CSF, a readily accessible resource for biomarker development pipelines (van Gool and Hendrickson, 2012). Therefore, the mass spectrometry data presented here can be used as a resource to establish future quantitative targeted searches of potential amygdaloid protein biomarkers in CSF by multiple reaction monitoring assays (Lehnert et al., 2012; Picotti and Aebersold, 2012).

## Concluding remarks

Taken together, our results provide a broad functional analysis of 1814 nonredundant human amygdaloid proteins, being the first step toward the complete characterization of this brain substructure. Our in-depth proteomic analysis contributes to the repertoire of the human brain proteome, providing fundamental information for the recently officially launched Human Proteome Project (HPP) (Legrain et al., 2011), designed to map the entire human protein set (Paik et al., 2012). Due to that amygdala is anatomically well-demarcated area with more than 10 different cytoarchitectonic nuclei (Sah et al., 2003), the development of specific isolation and purification protocols of single-cell types together with novel developments in shotgun proteomic approaches (Altelaar et al., 2013) would allow to explore the transcriptome and proteome profiling of each amygdaloid nucleus individually, increasing the molecular knowledge of the amygdaloid complex.

## Conflict of interest statement

The authors declare that the research was conducted in the absence of any commercial or financial relationships that could be construed as a potential conflict of interest.

## Abbreviations

MS: Mass spectrometry
MS/MS: Tandem mass spectrometry
RP-LC: Reverse Phase Liquid-Chromatography
2-DE: bidimensional electrophoresis
IEF: isoelectric focusing
pI: isoelectric point
HPLC: High Performance Liquid Chromatography
CSF: cerebrospinal fluid.
