# Supplementary material for: Applying mass spectrometry-based qualitative proteomics to human amygdaloid complex
Source: Front Cell Neurosci. 2014 Mar 20;8:80. doi: 10.3389/fncel.2014.00080 (PMC3960493; doi:10.3389/fncel.2014.00080)
Supplement: Supplementary file 1 [file Presentation1.PPT]

## Slide 1
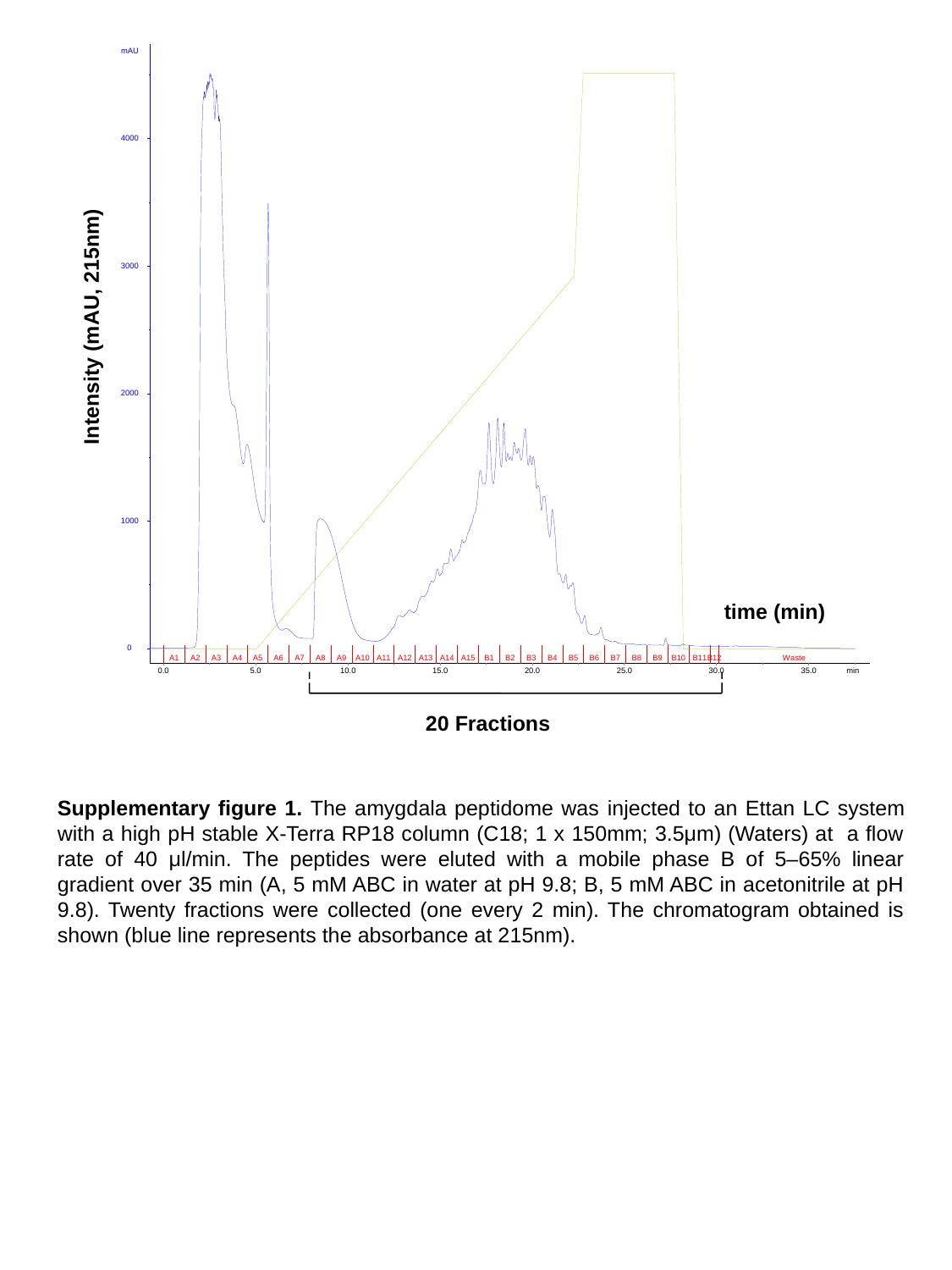

Intensity (mAU, 215nm)
time (min)
20 Fractions
Supplementary figure 1. The amygdala peptidome was injected to an Ettan LC system with a high pH stable X-Terra RP18 column (C18; 1 x 150mm; 3.5μm) (Waters) at a flow rate of 40 μl/min. The peptides were eluted with a mobile phase B of 5–65% linear gradient over 35 min (A, 5 mM ABC in water at pH 9.8; B, 5 mM ABC in acetonitrile at pH 9.8). Twenty fractions were collected (one every 2 min). The chromatogram obtained is shown (blue line represents the absorbance at 215nm).

## Slide 2
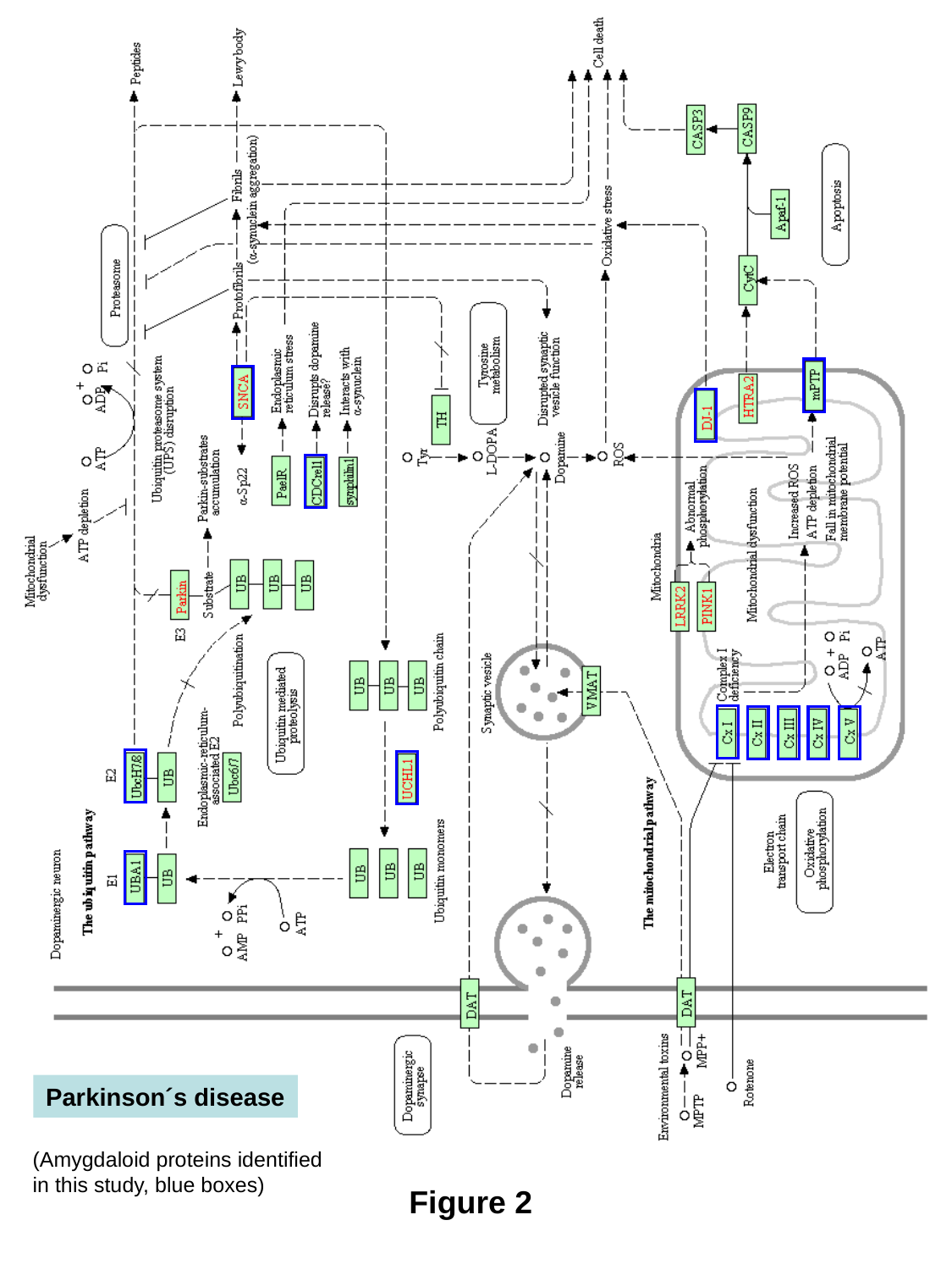

Parkinson´s disease
(Amygdaloid proteins identified
in this study, blue boxes)
Figure 2

## Slide 3
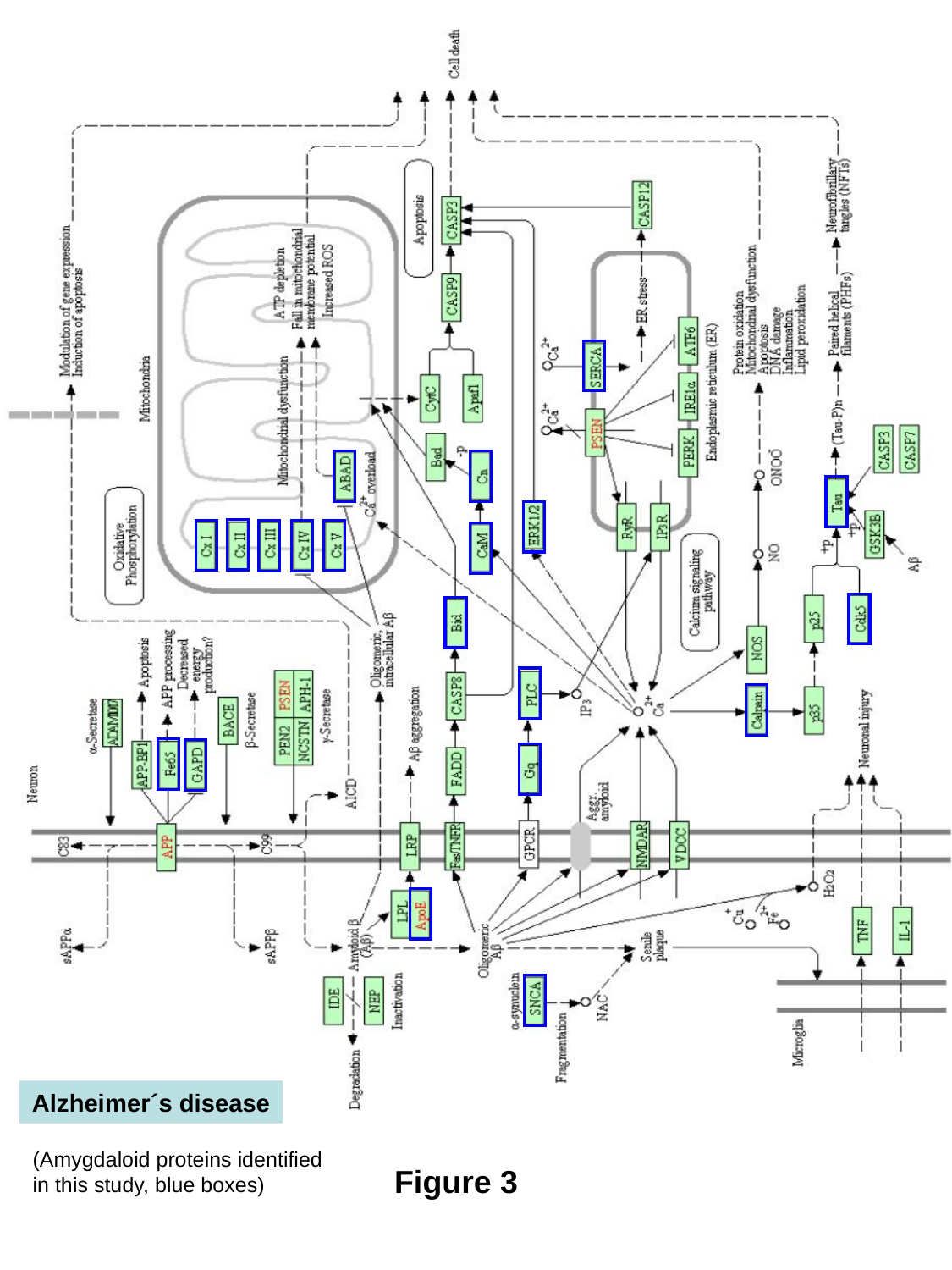

Alzheimer´s disease
(Amygdaloid proteins identified
in this study, blue boxes)
Figure 3

## Slide 4
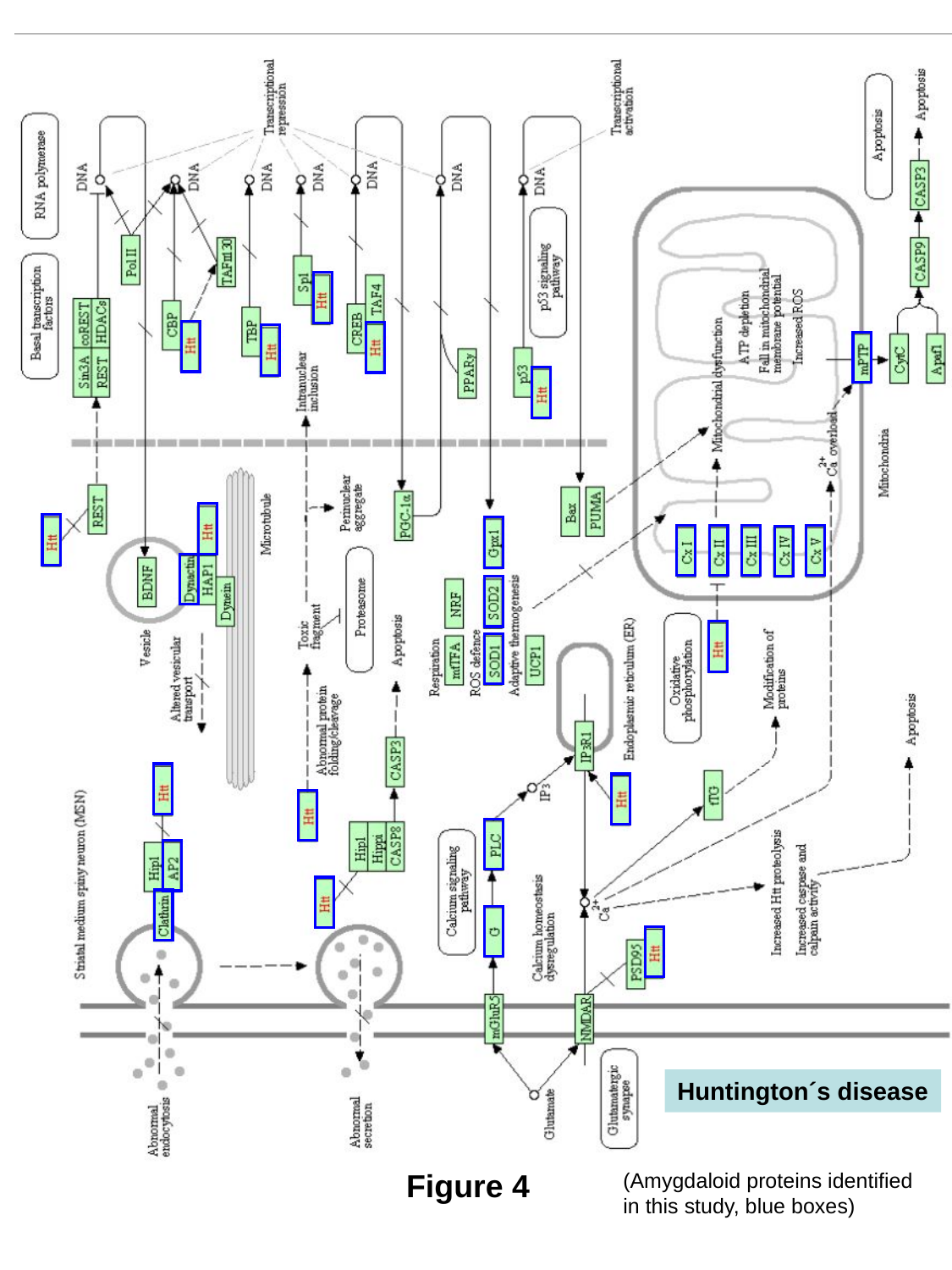

Huntington´s disease
Figure 4
(Amygdaloid proteins identified
in this study, blue boxes)
